# Polydatin Alleviates Small Intestine Injury during Hemorrhagic Shock as a SIRT1 Activator

**DOI:** 10.1155/2015/965961

**Published:** 2015-08-02

**Authors:** Zhenhua Zeng, Zhongqing Chen, Siqi Xu, Rui Song, Hong Yang, Ke-seng Zhao

**Affiliations:** ^1^Department of Critical Care Medicine, Nanfang Hospital, Southern Medical University, Guangzhou 510515, China; ^2^Guangdong Key Lab of Shock and Microcirculation Research, Department of Pathophysiology, Southern Medical University, Guangzhou 510515, China

## Abstract

*Objective*. To evaluate the role of SIRT1 in small intestine damage following severe hemorrhagic shock and to investigate whether polydatin (PD) can activate SIRT1 in shock treatment. *Research Design and Methods*. The severe hemorrhagic shock model was reproduced in Sprague Dawley rats. *Main Outcome Measures*. Two hours after drug administration, half of the rats were assessed for survival time evaluation and the remainder were used for small intestinal tissue sample collection. *Results*. Bleeding and swelling appeared in the small intestine with epithelial apoptosis and gut barrier disturbance during hemorrhagic shock. SIRT1 activity and PGC-1*α* protein expression of the small intestine were decreased, which led to an increase in acetylated SOD2 and decreases in the expression and activity of SOD2, resulting in severe oxidative stress. The decreased SIRT1 activity and expression were partially restored in the PD administration group, which showed reduced intestine injury and longer survival time. Notably, the effect of PD was abolished after the addition of Ex527, a selective inhibitor of SIRT1. *Conclusions*. The results collectively suggest a role for the SIRT1-PGC-1*α*-SOD2 axis in small intestine injury following severe hemorrhagic shock and that PD is an effective SIRT1 activator for the shock treatment.

## 1. Introduction

Prolonged resuscitation may induce ischemia/reperfusion (I/R) injury because of the burst of oxygen free radicals and subsequent mitochondrial injury [1]. Gut injury and subsequent loss of gut barrier function can lead to systemic inflammatory response syndrome and subsequent multiple organ dysfunction syndrome [2]. There is a need for alternative methods to treat shock-induced organ damage, especially in the gut. However, the mechanism underlying gut damage during severe shock remains uncertain.

Mitochondrial respiration is the major source of ATP, which is indispensable for maintaining cellular integrity and cellular function performance. Oxygen free radicals are also endogenously produced as a by-product of the mitochondrial electron transport systems (complexes I and III), and mitochondria have their own radical scavenging system to neutralize these radicals [3]. An imbalance between radical production and scavenging ability is thought to lead to mitochondrial injury [4]. Thus, a drug that scavenges mitochondria-origin oxygen free radicals may be an effective treatment for shock.

It is reported that peroxisome proliferator-activated receptor-gamma coactivator-1*α* (PGC-1*α*) is a powerful controller of cell metabolism and ensures the balance between the production and scavenging of prooxidant molecules by coordinating mitochondrial biogenesis [5] and the gene expression of antioxidants such as superoxide dismutase-2 (SOD2) [6]. Moreover, the activity [7–9] and expression [7] of PGC-1*α* can be inhibited by Sirtuin 1 (SIRT1), an enzyme that catalyzes deacetylation of acetyl-lysine residues of proteins such as p53 and FOXO3a [10]. Through NAD^+^-dependent deacetylase, SIRT1 has been reported to have an important role in cell proliferation, differentiation, senescence, apoptosis, metabolism, and oxidative stress resistance [11].

Resveratrol, a natural polyphenolic compound, was shown to improve mitochondrial function through activating SIRT1 and PGC-1*α* [12]. The effect of resveratrol on SIRT1 signaling has also been tested in prolonged life span [12], Parkinson's disease [13, 14], obesity [15], amyotrophic lateral sclerosis [16], diabetic milieu [17], and trauma-hemorrhage shock [18, 19]. Its analog polydatin (PD), which is also known as resveratrol glucoside, is a monocrystalline drug that can be isolated from the traditional Chinese herb* Polygonum cuspidatum*. The molecular composition of PD is 3, 4′, 5-trihydroxystilbene-3-monoglucoside. We previously demonstrated that PD can protect arterial smooth muscle and neuronal cells against mitochondrial dysfunction after severe ischemia-reperfusion injury in a rat model of hemorrhagic shock; notably, its effect was even better than that of resveratrol [20, 21]. However, the effect of PD on intestinal cell mitochondria following shock-induced gut injury is not known. In this study, we evaluated SIRT1-PGC-1*α* axis activity in small intestine tissue subjected to severe hemorrhagic shock and found a downstream target of this pathway. In addition, we investigated the protective effect of PD on small intestine injury and clarified its relationship with the SIRT1 pathway.

## 2. Methods

### 2.1. Reagents and Antibodies

PD and its specific vehicle (ethanol 70%, propylene glycol 20%, and NaHCO_3_ 10%) were supplied by Neptunus Co. (Shenzhen, Guangdong, China); its purity was over 99.5%. Antibodies against Bcl-2, Bax, SOD2, and acetylated SOD2 were obtained from Epitomics (Burlingame, CA, USA). The SOD activity assay kit was obtained from Dojindo Molecular Technology Inc. (Gaithersburg, MD, USA). Anti-SIRT1 and PGC-1*α* antibodies were from Santa Cruz Biotechnology (Santa Cruz, CA, USA). The SIRT1 activity assay kit was from Abcam (Cambridge, UK). The reduced glutathione/oxidized glutathione (GSH/GSSG) and catalase (CAT) assay kits were from Beyotime Biotech (Beijing, China). All other chemicals were from Sigma (St. Louis, MO, USA).

### 2.2. Establishment of the Rat Hemorrhagic Shock Model

This study was carried out in strict accordance with the recommendations of the Guide for the Care and Use of Laboratory Animals of the U.S. National Institutes of Health and was approved by the Committee on Ethics in Animal Experiments of the University of Southern Medical University. Adult specific pathogen free (SPF) female Sprague Dawley (SD) rats, weighing 180–220 g (7-8 weeks old), were obtained from the Laboratory Animal Center (Southern Medical University, Guangzhou, China) and were housed in metabolic cages under controlled environmental conditions (25°C and a 12 h light/dark cycle). Animals had free access to standard rat pellet food and tap water. All efforts were made to minimize animal's suffering and to reduce the number of animals used. Sixty-four rats were anesthetized with a mixture of 13.3% urethane and 0.5% chloralose *α* (0.65 mL/100 g body weight); then the rats were subjected to hemorrhagic shock for 120 min followed by reinfusion of whole shed blood (with small modifications) as previously described [21]. Briefly, after implantation of PE-50 catheters in left femoral arterial and left femoral venous passages, the mean artery pressure (MAP) was recorded using PowerLAB registration equipment (AD Instruments, Sydney, Australia). The rats were bled through a syringe to obtain an MAP of 30 mmHg within 10 min, which was maintained for the next 2 h by withdrawal or reinfusion of stored blood. Then, PD, vehicle, or PD/Ex527 (Ex527 is a SIRT inhibitor) was intravenously administered within 10 min; after that, the whole shed blood was reinfused within 10 min. Then the animals were randomly divided into four groups: (1) the control (sham) group, in which the rats were anesthetized and implanted with PE-50 catheters in left femoral artery and vein as it has been done in other groups but without any other treatments; (2) the vehicle group, in which the rats were subjected to hemorrhage shock to maintain the MAP at 30 mmHg for 120 min, followed by administration of the vehicle (0.3 mL) and shed blood infusion; (3) the PD group, in which the rats were subjected to shock for 120 min, followed by administration of PD (30 mg/kg) dissolved in 0.3 mL vehicle and shed blood infusion (the dose of PD administration was based on our previous studies [20, 21]); and (4) the PD/Ex527 group, in which the rats were subjected to shock for 120 min, followed by administration of PD (30 mg/kg) and Ex527 (5 mg/kg) [22] dissolved in 0.3 mL vehicle and shed blood infusion.

Thirty-two rats (8 in each group) were euthanized by cervical dislocation 2 h after the shed blood was reinfused. Then, 1.0 mL arterial blood was collected and centrifuged at 1000 ×g for 15 min at 4°C and then was stored at −80°C for inflammatory cytokine measurement. A laparotomy was performed, and after gross observation of the small intestine, 10 cm of ileum from 10 cm distal to the ligament of Treitz was carefully removed. One part of the tissue was homogenized for protein extraction, and the remained was fixed in neutral-buffered formalin.

### 2.3. Histopathological Analysis and Chiu Scoring

The formalin-fixed specimens were embedded in paraffin, cut into 4 *μ*m thick transverse sections, and stained with hematoxylin and eosin (H&E) to assess epithelial morphology under a light microscope (Axio Imager Z2, Carl Zeiss Inc., Oberkochen, Germany). The intestinal mucosal injury was evaluated with the Chiu scoring system [23] in a single blinded fashion, which was graded as Grade 0: normal mucosa; Grade 1: subepithelial detachments at the tips of villi with capillary congestion; Grade 2: subepithelial detachments exerting a moderate upward push on the mucosal epithelium; Grade 3: large subepithelial detachments exerting a massive upward push on the mucosal epithelium along the villi, and a few denuded villus tips are observed; Grade 4: the villi are denuded to the level of the lamina propria and dilated capillaries are observed; and Grade 5: ulceration, disintegration of lamina propria, and hemorrhage. Ten fields for each sample were observed, and the average score was recorded as the small intestinal tissue pathology score.

### 2.4. Western Blot Analysis

Small intestinal tissue samples were lysed in radioimmunoprecipitation assay buffer, and proteins were collected after centrifugation and mixed with 5x sodium dodecyl sulfate (SDS) sample buffer. The samples were separated by SDS-polyacrylamide gel electrophoresis (PAGE) using 8–12% acrylamide gels and then transferred to polyvinylidene fluoride (PVDF) membranes (Millipore, Billerica, MA, USA). After incubation with primary antibodies (against Bax, Bcl-2, SOD2, acetylated-SOD2, SIRT1, and PGC-1*α*) and secondary antibodies, protein bands were detected using chemiluminescence detection reagents (Millipore).

### 2.5. Detection of Epithelial Cell Apoptosis

Apoptosis assays of intestinal tissues sections were assessed using terminal deoxynucleotidyl transferase dUTP nick-end labeling (TUNEL) assays (DeadEnd Fluorometric TUNEL System; Promega, Madison, WI, USA). Briefly, the TdT reaction mixture was added to the tissue sections and incubated for 60 min at 37°C in a humidified chamber after the tissue sections were prepared. Then, the sections were incubated sequentially with rTdT incubation buffer and DAPI after they were washed with phosphate-buffered saline (PBS). Finally, the slides were left to develop until a light brown background appeared. Morphological nuclear changes were observed under a confocal microscope (LSM 780, Carl Zeiss Inc.). The apoptotic cells were counted in 10 random high-power fields (HPF, 300 cells each), and a total of 1500 epithelial cells were counted. The positive cells that stained brown were counted as apoptotic. Data are expressed as the number of apoptotic cells/HPF (400x).

### 2.6. Detection of Oxidative Stress-Related Enzyme Production

Total GSH, GSSG/GSH ratio, and CAT activity in fresh small intestine tissue homogenate were evaluated using commercially available kits according to the manufacturer's instructions and standard methods. The optical density at 412 nm was determined for GSH and GSSG by a Microplate Reader (SpectraMax M5), and the concentrations of these two enzymes were calculated. For CAT, the optical density was determined at 520 nm on a UV spectrophotometer. The relative values of CAT compared to the control group are presented.

The SOD2 activity assay was carried out with the SOD assay kit using water-soluble tetrazolium salt (WST-1) as a substrate, following the manufacturer's instructions (Dojindo Molecular Technology Inc.). Briefly, the total SOD activity in each fraction was measured by inhibition of the rate of WST-1 reduction. SOD2 activity was measured by adding 1 mmol/L potassium cyanide to each fraction to inactivate Cu/ZnSOD. SOD2 activity is expressed as units per milligram of protein (1 unit was defined as the amount of enzyme that inhibited WST-1 reduction by 50%). The relative SOD2 activity (compared to that of the normal control) values are shown.

### 2.7. SIRT1 Activity Assay

SIRT1 enzymatic activity was measured using a commercial kit (ab156065; Abcam). According to the manufacturer's directions, fresh intestinal tissues were immunoprecipitated (Immunoprecipitation Kit; Proteintech, Chicago, IL, USA) with anti-SIRT1 antibody (Santa Cruz Biotechnology). Then, the reaction mixture containing fluoro-substrate peptide solution and protein A agarose beads was added, and NAD-dependent deacetylase activity was measured based on fluorescence intensity at 1-2 min intervals at Ex/Em = 350/460 nm in SpectraMax M5. The activity is presented as the relative value compared to the control group.

### 2.8. Serum Inflammatory Cytokine Measurement

The frozen serum was thawed on ice. Levels of interleukin- (IL-) 1*β*, IL-6, and tumor necrosis factor (TNF)-*α* were detected by enzyme-linked immunosorbent assay (ELISA) according to the manufacturer's instructions (Elabscience Biotechnology Co., Ltd., Wuhan, China).

### 2.9. Survival Time Analysis

After treatment and suturing, the survival time was recorded for 32 animals (8 in each group). All animals had ad libitum access to food and water. Apnea >1 min was considered to indicate death. Animals that survived longer than 48 h were euthanized by cervical dislocation.

### 2.10. Statistical Analysis

The median survival times were analyzed with Kaplan-Meier plots and compared using the log-rank test with GraphPad Prism 6.0.3 (GraphPad Software Inc., La Jolla, CA, USA). The other data was expressed as mean ± standard deviation and was analyzed by SPSS software (SPSS Inc., Chicago, IL, USA). The homogeneity of variance test (Levene's test) was used to assess whether groups had equal variances. ANOVA was performed when Levene's test indicated homogeneity of variance (*P* > 0.1). When ANOVA showed significant differences among groups (*P* < 0.05), Tukey's HSD multiple-comparison test was performed. When equal variances were not assumed (based on Levene's test; *P* < 0.1), robust tests of equality of means were used by Dunnett's T3 post hoc comparisons. Values were considered statistically significant at *P* < 0.05.

## 3. Results

### 3.1. Small Intestine Injury during Severe Hemorrhagic Shock

#### 3.1.1. Small Intestine Histopathology

**Figure 1:**
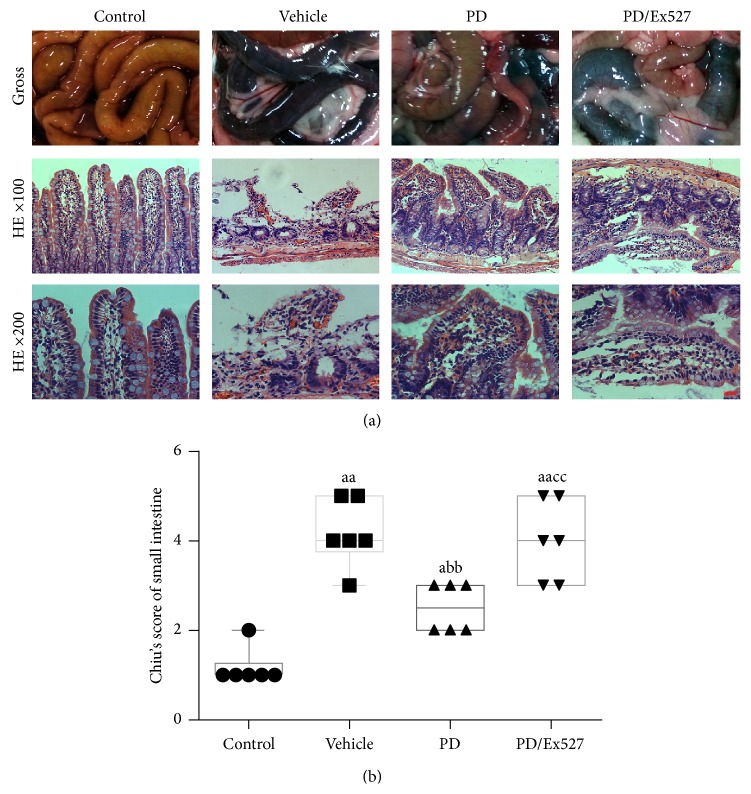
Gross morphologic alterations and histopathologic changes of the small intestine in severe shock rats. (a) Obvious bleeding and swelling appeared in both the vehicle and PD/Ex527 groups, but these were reduced in the PD group. Histologic lesions of the rat small bowel are shown in the bottom two rows. Shortened, broadened villi and extensive denudation of the villus epithelium were seen in the vehicle and PD/Ex527 groups, with mild broadening of the villi and disruption of villus epithelium in the PD group. (b) Chiu injury score of small intestine (*n* = 6 rats per group). ^a^ *P* < 0.05, ^aa^ *P* < 0.01 compared with the control group, ^bb^ *P* < 0.01 compared with the vehicle group, and ^cc^ *P* < 0.01 compared with the PD group. HE, hematoxylin-eosin staining; PD, polydatin.

First, we determined if small intestine injury took place during severe shock by evaluating intestine histopathology. Hemorrhagic shock for 2 h followed by reperfusion for another 2 h resulted in obvious bleeding and swelling in the small intestine of the vehicle group (Figure 1(a)). Moreover, marked villous stroma broadening, focal necrosis, and some epithelial cell detachment accompanied by marked edema and congestion were observed on histologic evaluation with H&E staining (Figure 1(a)). The Chiu score was also increased (Figure 1(b)). These results collectively indicated that severe intestine injury occurred in severe shock rats.

#### 3.1.2. Small Intestine Cell Apoptosis

**Figure 2:**
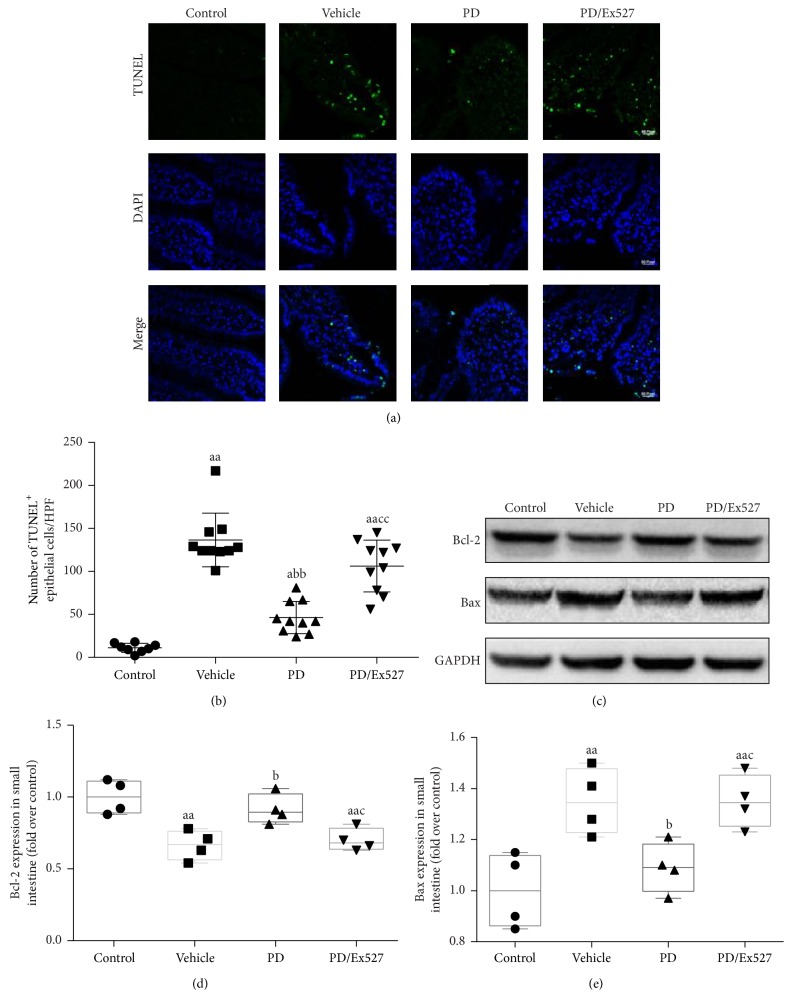
TUNEL apoptosis staining and Bcl-2 and Bax protein levels in the small intestine epithelial following severe shock. (a) TUNEL staining (original magnification ×400). (b) Statistical analysis of TUNEL results (*n* = 8–10). (c) Representative western blotting for apoptosis-related protein (Bcl-2 and Bax). (d-e) Densitometry analysis of Bcl-2 and Bax (*n* = 4 per group). ^a^ *P* < 0.05, ^aa^ *P* < 0.01 compared with the control group; ^b^ *P* < 0.05, ^bb^ *P* < 0.01 compared with the vehicle group; ^c^ *P* < 0.05, ^cc^ *P* < 0.01 compared with the PD group. DAPI, 4′,6-diamidino-2-phenylindole; TUNEL, terminal deoxynucleotidyl transferase dUTP nick end labeling; HPF, high power field; Bcl-2, B-cell lymphoma-2; Bax, Bcl-2-associated X protein; PD, polydatin.

Next, we determined cell apoptosis in the small intestine. The number of TUNEL^+^ cells in the vehicle group increased over 10-fold compared to the normal (control) group (*P* < 0.01, Figures 2(a) and 2(b)). Expression of the proapoptosis protein Bax was increased, and the antiapoptosis protein Bcl-2 was reduced (*P* < 0.01 for all, Figures 2(c)–2(e)).

#### 3.1.3. Small Intestine Oxidative Stress

**Figure 3:**
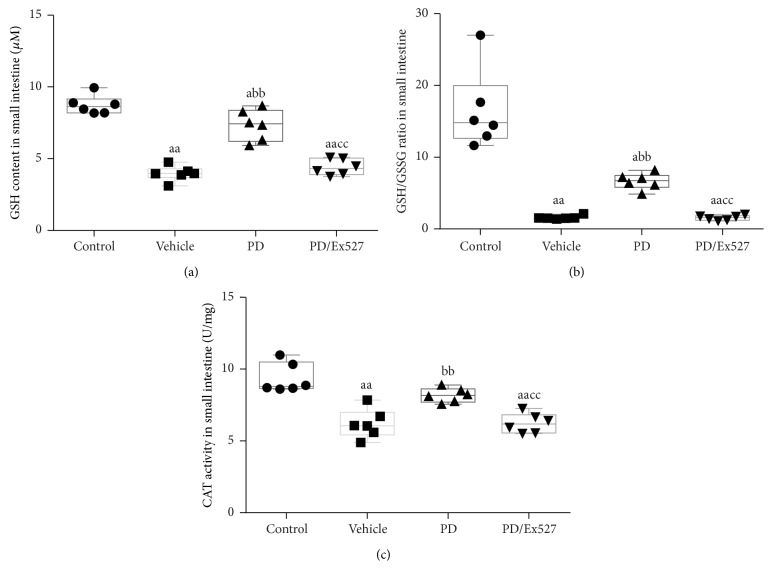
Oxidative stress-related indexes in the small intestine following severe hemorrhagic shock. (a) Reduced glutathione (GSH) content, (b) GSH/GSSG ratio, and (c) catalase (CAT) activity were analyzed in small intestine homogenate (*n* = 6 per group). ^a^ *P* < 0.05, ^aa^ *P* < 0.01 compared with the control group; ^bb^ *P* < 0.01 compared with the vehicle group; ^cc^ *P* < 0.01 compared with the PD group. GSH, reduced glutathione; GSSG, oxidized glutathione; CAT, catalase; PD, polydatin.

To evaluate the general oxidative stress condition of small intestine tissue, GSH content, GSH/GSSG ratio, and CAT activity in homogenate were determined. Compared with the control group, the values of the three markers were significantly decreased in the vehicle group (*P* < 0.01 for all, Figure 3).

### 3.2. SIRT1-PGC-1*α*-SOD2 Pathway in the Pathogenesis of Small Intestine Damage

#### 3.2.1. SOD2 Protein Expression and Activity

**Figure 4:**
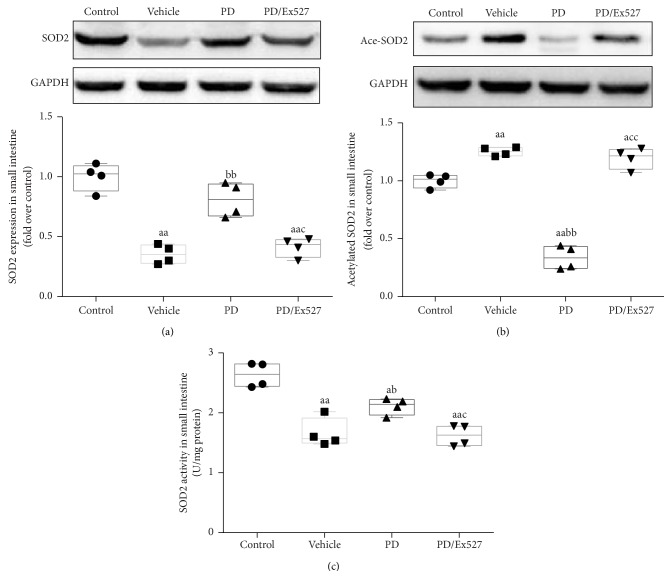
Protein expression, acetylation, and activity of SOD2 in small intestinal tissue following severe hemorrhagic shock. (a) Representative western blotting bands of SOD2 protein and relative gray values (*n* = 4 per group). (b) Acetylated SOD2 (Ace-SOD2) level determination (*n* = 4 per group). (c) SOD2 activity of small intestinal homogenate (*n* = 4 per group). ^a^ *P* < 0.05, ^aa^ *P* < 0.01 compared with the control group; ^b^ *P* < 0.05, ^bb^ *P* < 0.01 compared with the vehicle group; ^c^ *P* < 0.05, ^cc^ *P* < 0.01 compared with the PD group. SOD2, superoxide dismutase 2; Ace-SOD2, acetylated superoxide dismutase 2; GAPDH, glyceraldehyde 3-phosphate dehydrogenase; PD, polydatin.

Severe small intestine injury was found in severe hemorrhagic shock rats, and mitochondria-originated oxygen free radicals were the main source of oxidative stress. We therefore determined if SOD2, an antioxidative stress enzyme located in mitochondria, was damaged in rats subjected to intestinal injury. Interestingly, SOD2 protein expression in small intestine tissue homogenate was decreased by 64.7% ± 9.8% (*P* < 0.01, Figure 4(a)). Based on the deacetylase role of SIRT1, we assumed that the level of acetylated SOD2 may be modulated. As expected, the level of acetylated SOD2 was significantly increased (*P* < 0.01, Figure 4(b)). In contrast, SOD2 activity was significantly decreased by 73% ± 9% (*P* < 0.01, Figure 4(c)). These results indicated that both the reduced SOD2 protein expression and its increased acetylation might account for the decreased SOD2 activity.

#### 3.2.2. PGC-1*α* and SIRT1 Proteins Expression and SIRT1 Activity

**Figure 5:**
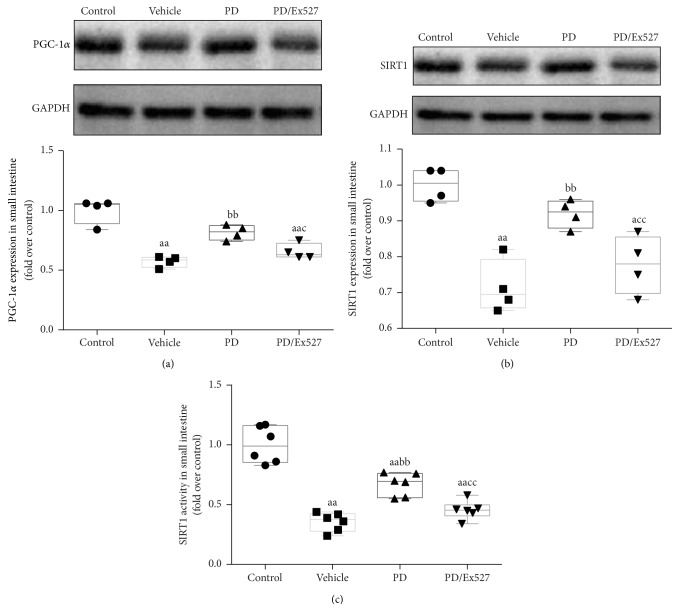
Protein expression of PGC-1*α* and protein expression and activity of SIRT1 in small intestine tissue following severe shock. (a) PGC-1*α* and (b) SIRT1 proteins expression in small intestine tissue (*n* = 4 per group). (c) Relative SIRT1 activity of small intestine tissue (*n* = 6 per group). ^a^ *P* < 0.05, ^aa^ *P* < 0.01 compared with the control group; ^bb^ *P* < 0.01 compared with the vehicle group; ^c^ *P* < 0.05, ^cc^ *P* < 0.01 compared with the PD group. PGC-1*α*, peroxisome proliferator-activated receptor-gamma coactivator-1 alpha; GAPDH, glyceraldehyde 3-phosphate dehydrogenase; PD, polydatin.

Given the regulatory effect of PGC-1*α* and SIRT1 on SOD2 and the interaction between PGC-1*α* and SIRT1, we hypothesized that SOD2 might be modulated by the “PGC-1 *α*-SIRT1 axis.” We therefore measured PGC-1*α* protein expression and SIRT1 protein expression and activity. Compared with the control group, the relative protein expressions of PGC-1*α* and SIRT1 were decreased by 42.5% ± 5.7% and 23% ± 7.1% in the vehicle group, respectively (*P* < 0.01 for all, Figures 5(a) and 5(b)). As expected, the relative activity of SIRT1 was decreased to less than half that of the control group (*P* < 0.01, Figure 5(c)). These results showed that both SIRT protein expression and activity were reduced in the small intestine of rats subjected to severe shock.

### 3.3. PD Upregulated SIRT1 and Reduced Small Intestine Injury in Severe Shock

Given the effects of PD on multiple organ protection and the fact that PD possesses a similar structure to resveratrol, we supposed that PD could directly activate SIRT1. To test this hypothesis, we performed experiments with Ex527, a selective SIRT1 inhibitor.

**Figure 6:**
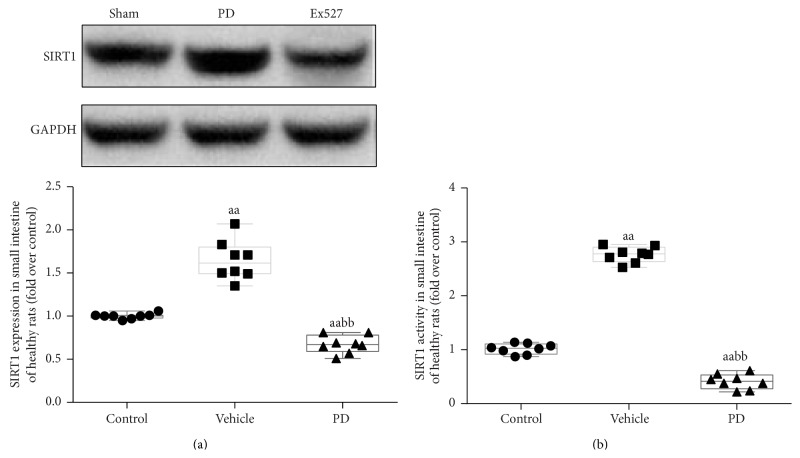
SIRT protein expression and activity in the small intestine tissue of healthy rats. After 7 days' administration of PD or Ex527 (a selective SIRT1 inhibitor), levels of both the SIRT1 protein (a) and activity (b) were markedly increased in the PD group but were all reduced in the Ex527 group (*n* = 8 per group). ^aa^ *P* < 0.01 compared with the sham group; ^bb^ *P* < 0.01 compared with the PD group. SIRT1, silent information regulator 1; GAPDH, glyceraldehyde 3-phosphate dehydrogenase; PD, polydatin.

We first evaluated the effects of PD and Ex527 on intestine in sham-operated rats. We found that after intraperitoneal administration of the two agents at experimental dosage for 7 days, SIRT1 protein level and activity were markedly increased in the PD administration group but significantly decreased in the Ex527 group (*P* < 0.01 for both groups versus sham, Figure 6).

We then tested SIRT1 protein expression and activity and measured PGC-1*α* protein expression in small intestine homogenate following severe hemorrhagic shock and reperfusion. Compared with vehicle, the relative protein expressions of SIRT1 and PGC-1*α* were increased in the PD group. Similarly, the relative activity of SIRT1 was increased nearly twofold over the vehicle group (*P* < 0.01, Figure 5(c)). However, Ex527 treatment almost abolished the activating effect of PD on SIRT1 and PGC-1*α* (Figure 5), which further supported the hypothesis that the PD protective effect may involve SIRT1 signaling.

The therapeutic effects of PD on small intestine oxidative stress, apoptosis, and histological changes were determined. As expected, PD administration elevated SOD2 protein expression but reduced the level of acetylated SOD2 (all *P* < 0.01, Figures 4(a) and 4(b)). Importantly, PD increased SOD2 activity to over twofold that of the vehicle group (*P* < 0.01, Figure 4(c)). In addition, PD treatment significantly restored the GSH content, GSH/GSSG ratio, and CAT activity (all *P* < 0.01 versus vehicle group, Figure 3). In agreement with the oxidative stress determination results, fewer TUNEL^+^ cells were found in the PD group (*P* < 0.01 versus vehicle group, Figures 2(a) and 2(b)), and expression of the pro- and antiapoptosis proteins Bax and Bcl-2 decreased and increased, respectively (*P* < 0.05 for Bax and *P* < 0.01 for Bcl-2, Figure 2(c)–2(e)). Moreover, PD administration reduced the degrees of bleeding and swelling, with only slight broadening of the villous stroma and minimal epithelial cell detachment. It also decreased the Chiu score (Figures 1(a) and 1(b)). However, after Ex527 was added, the protective effects of PD against oxidative stress, apoptosis, and histological changes were all diminished (Figures 1–3).

### 3.4. PD Improved Rat General Condition during Severe Hemorrhagic Shock

**Figure 7:**
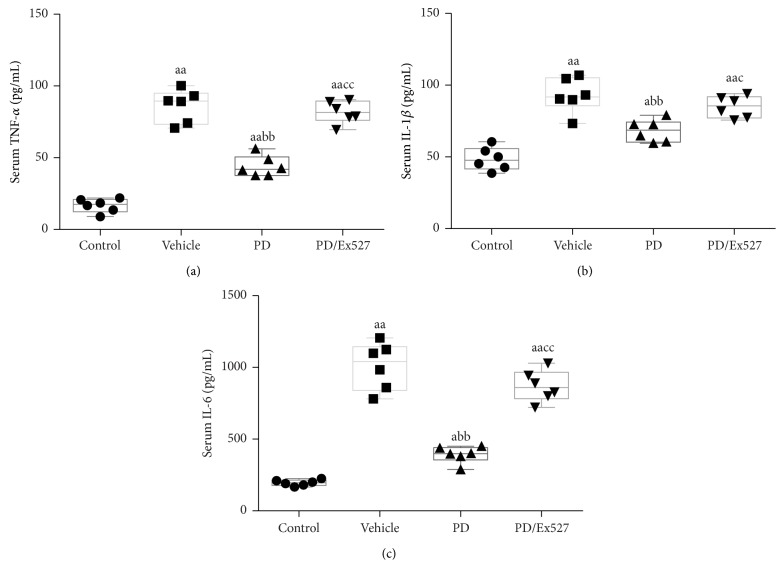
Serum inflammatory cytokine measurement following severe hemorrhagic shock (*n* = 8 rats). ^a^ *P* < 0.05, ^aa^ *P* < 0.01 compared with the control group; ^bb^ *P* < 0.01 compared with the vehicle group; ^c^ *P* < 0.05, ^cc^ *P* < 0.01 compared with the PD group. TNF-*α*, tumor necrosis factor-*α*; IL-1*β*, interleukin-1 beta; IL-6, interleukin-6; PD, polydatin.

**Figure 8:**
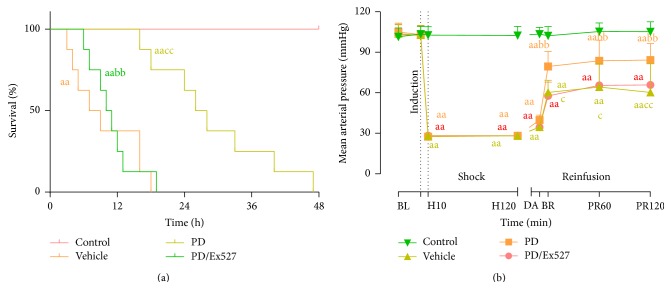
Mean arterial pressure and survival time of rats subjected to hemorrhagic shock and reperfusion. (a) Survival analysis was assessed with the Kaplan-Meier method and log-rank tests (*n* = 8 rats per group). (b) Time course of MAP during 120 minutes of hemorrhagic shock followed by 120 minutes of observation (*n* = 8 rats per group). ^aa^ *P* < 0.01 compared with the control group; ^bb^ *P* < 0.01 compared with the vehicle group; ^c^ *P* < 0.05, ^cc^ *P* < 0.01 compared with the PD group. BL, baseline; DA: drug administration; H, hemorrhage; R, reinfusion; PR, postreinfusion; PD, polydatin.

Finally, we evaluated the effect of PD on general animal condition including the systemic inflammatory response, MAP, and survival time. We found that 2 h after shed blood infusion, serum proinflammatory cytokine levels were elevated (Figure 7), and MAP was sharply decreased (Figure 8(b)). Consequently, all the rats in the vehicle group died within 48 h (Figure 8(a) and Table 1). Notably, PD administration significantly mitigated the inflammatory response, elevated MAP 2 h after shed blood reinfusion, and prolonged the survival time of shock rats. Five of eight rats survived for over 24 h, with a median survival time of 27 h (*P* < 0.01 versus vehicle group, Figure 8(a) and Table 1). As expected, Ex527 treatment blocked the beneficial effects of PD on system inflammatory mitigation, MAP, and survival time (*P* < 0.01, Figure 8(a) and Table 1).

## 4. Discussion

The major findings of this study are as follows. First, we confirmed that SIRT1 protein level and activity were decreased in the setting of intestinal injury after severe hemorrhagic shock and reperfusion, and these changes may be related to alterations in the SIRT1-PGC-1*α*-SOD2 axis signaling pathway. Second, we found that PD administration effectively protected against shock-induced intestinal injury. The protective effect of PD may be associated with its SIRT1 activating effect. To the best of our knowledge, this is the first report demonstrating PD as a new activator of SIRT1, which may lay an important experimental basis for the future clinical application of PD.

SIRT1 is one of seven mammalian homologs of Sir2 that catalyzes NAD^+^-dependent protein deacetylation, which was originally described as a factor regulating longevity, apoptosis, and DNA repair [9]. In a variety of organisms, SIRT1 regulates gene expression through the deacetylation of histone, transcription factors, and lysine residues of other modified proteins including several metabolic and endocrine signal transcription factors such as PPAR*γ*, P53, FOXO3a, and PGC-1*α* [9, 24, 25]. PGC-1*α*, a transcriptional coactivator, is a potent stimulator of mitochondrial biogenesis and respiration. Increasing PGC-1*α* levels dramatically protect cultured neural cells from oxidative stressor-mediated death [12]. Also, it was reported that, in a trauma and hemorrhagic shock model, SIRT1 and PGC-1*α* protein expressions were reduced [18, 19, 26] and SIRT1 activity was decreased [18] in the left ventricular tissue of rats. The effect of PGC-1*α* is related to its coinduction with SOD2 enzyme [25]. SOD is the main enzymatic defense against superoxide anions. SOD2, a member of the iron/manganese superoxide dismutase family located in mitochondria, detoxifies the superoxide anion, thus converting it into H_2_O_2_ and water [27]. In this study, SIRT1 protein expression and activity and PGC-1*α* protein expression were decreased following severe shock and shed blood reperfusion, suggesting that the SIRT1-PGC-1*α* axis may be inhibited in damaged small intestine. As the downstream target of the SIRT1-PGC-1*α* axis, SOD2 activity was decreased to 37 ± 9% of the control value, possibly due to lower SOD2 expression and increased SOD2 acetylation. On the one hand, decreased SIRT1 activity downregulates PGC-1*α* protein, thus decreasing SOD2 protein expression. On the other hand, in case of the decreased SIRT1 activity, the deacetylation regulation effect was weakened, and acetylated SOD2 protein expression was increased, resulting in reduced antioxidative ability. Subsequently, the reduced SOD2 activity caused the decreased GSH content and GSH/GSSG ratio in the small intestine, promoted oxidative stress, and led to mitochondria-mediated apoptosis and small intestine injury.

Interestingly, we previously showed that PD treatment could protect arterial smooth muscle and neuronal cells against shock-induced mitochondrial injury. Notably, PD possessed a stronger antioxidant property than its analog resveratrol [20, 21]. As an antioxidative agent, resveratrol can scavenge free radicals via its 4′-hydroxyl group; the phenoxyl radical delocalizes the unpaired electron [28]. This might also be how the antioxidant effects of PD (also named piceid) are mediated. Unlike resveratrol, the substitution of a hydroxyl for a glycoside group makes the radical more stable for steric hindrance and prevents its reaction with other PD molecules [28]. Recently, resveratrol has been shown to activate SIRT1 [29]. Subsequent reports revealed that resveratrol could restore SIRT1 protein expression [18, 19, 26] and activity [18] in the left ventricular tissue of rats following hemorrhagic shock. Moreover, resveratrol was proved to prolong the survival time of hemorrhagic shock rat in the absence of resuscitation, and resveratrol showed identical effect with SIRT1 activator SIRT1720 [30]. In this study, we hypothesized that PD can protect against intestine injury due to its effects on SIRT1 signaling. As expected, we found that PD administration effectively increased SIRT1 protein expression and activity in the intestine homogenate of normal control rats. Furthermore, PD treatment restored SIRT1 activity in small intestine during severe shock. The increased SIRT1 activity increased SOD2 protein content by upregulating PGC-1*α* protein expression and decreasing acetylated SOD2 levels via direct or indirect deacetylating effects, thus increasing SOD2 activity. This attenuated oxidative stress levels and reduced apoptosis. To provide additional evidence that PD impacts SIRT1 signaling, we introduced a special SIRT1 inhibitor (Ex527). We found that the benefits of PD treatment were abolished after Ex527 addition. Thus, we propose that PD, similar to resveratrol, activates SIRT1 and that protection against small intestine injury might rely on the SIRT1-PGC-1*α*-SOD2 axis. In addition to the attenuation of oxidative stress, PD treatment mitigated the systemic inflammatory response. Again, this effect was abolished by Ex527, indicating that the inflammatory inhibition of PD might be related to its SIRT1 activating effect.

Our study has some potential limitations. Firstly, survival time was only observed for 48 h since none of the animals in the vehicle and PD/Ex527 groups survived for longer. More importantly, we only studied a single PD dose. However, based on the dose-effect study in our previous work [21], we found that a 30 mg/kg dose of PD achieved better therapeutic effects than 15 mg/kg and had similar effects to 45 mg/kg. Secondly, we only observed a single time point 2 h after blood reinfusion; this was selected based on our earlier observation of severe mitochondrial dysfunction at that time [20, 21]. More time points should be assessed in future investigations. Thirdly, resveratrol was not included in this study because it previously showed an inferior therapeutic effect compared to PD [21]. However, the results might be more convincing if the two analogs were directly compared. Fourthly, only female rats with same age were used in this study. Some previous reports revealed that female rats were more resistant to decreased blood pressure [31–33]. In contrast, Liu et al. found that resuscitation effects on hemorrhagic shock rats had no sex difference. Nevertheless, the controls and treatment animals were treated identically to avoid the gender and the estrous cycle impact. Fifthly, we used urethane as an anesthetic drug in this study. Urethane was reported to possess strong inhibition action on the circulatory system, somehow exerting hypovolemic effect [34]. Also urethane might affect the mesenteric vasculature and prolong the clotting time [35]. However, as long as the controls and treatment animals are treated identically, this may not be a major concern for the study. Nevertheless, these results demonstrate that PD may be a promising candidate for the treatment of shock-induced intestine injury.

## 5. Conclusion

This study shows the important role of the SIRT1-PGC-1*α*-SOD2 axis in the genesis of small intestine injury following severe shock. In addition, our findings suggest that PD treatment exerts a profound protective effect against gut injury in the setting of severe shock. This protection appears to be largely due to PD's ability to activate the SIRT1 pathway and attenuate oxidative stress.

## Figures and Tables

**Table 1:** Survival time and rate of rats following severe shock (mean ± SD).

Group	Weight (g)	Blood loss (mL/100 g)	Median survival time (h)	24 h survival rate
Control	205.1 ± 7.4	0	48	8/8
Vehicle	206.6 ± 6.3	3.2 ± 0.1	8^aa^	0/8^aa^
PD	209.1 ± 7.5	3.2 ± 0.1	27^bb^	5/8^bb^
PD/Ex527	204 ± 6.4	3.1 ± 0.1	10.5^aacc^	0/8^aacc^

^aa^ *P* < 0.01 compared with the control group; ^bb^ *P* < 0.01 compared with the vehicle group; ^cc^ *P* < 0.01 compared with the PD group.
